# Immune2vec: Embedding B/T Cell Receptor Sequences in ℝ*^N^* Using Natural Language Processing

**DOI:** 10.3389/fimmu.2021.680687

**Published:** 2021-07-22

**Authors:** Miri Ostrovsky-Berman, Boaz Frankel, Pazit Polak, Gur Yaari

**Affiliations:** ^1^ Bioengineering, Faculty of Engineering, Bar Ilan University, Ramat Gan, Israel; ^2^ Bar Ilan Institute of Nanotechnologies and Advanced Materials, Bar Ilan University, Ramat Gan, Israel

**Keywords:** biological sequence embedding, word2vec, NLP, BCR repertoire, computational immunology

## Abstract

The adaptive branch of the immune system learns pathogenic patterns and remembers them for future encounters. It does so through dynamic and diverse repertoires of T- and B- cell receptors (TCR and BCRs, respectively). These huge immune repertoires in each individual present investigators with the challenge of extracting meaningful biological information from multi-dimensional data. The ability to embed these DNA and amino acid textual sequences in a vector-space is an important step towards developing effective analysis methods. Here we present Immune2vec, an adaptation of a natural language processing (NLP)-based embedding technique for BCR repertoire sequencing data. We validate Immune2vec on amino acid 3-gram sequences, continuing to longer BCR sequences, and finally to entire repertoires. Our work demonstrates Immune2vec to be a reliable low-dimensional representation that preserves relevant information of immune sequencing data, such as n-gram properties and IGHV gene family classification. Applying Immune2vec along with machine learning approaches to patient data exemplifies how distinct clinical conditions can be effectively stratified, indicating that the embedding space can be used for feature extraction and exploratory data analysis.

## Introduction

Antibodies, the secreted form of BCRs, play a crucial role in the adaptive immune system, by binding specifically to pathogens and neutralizing their activity (1). The BCR repertoire in humans is estimated to include at least 10^11^ different BCRs, and potentially several orders of magnitude greater (2). High Throughput Sequencing (HTS) is a powerful platform that enables large-scale characterization of BCR repertoires (3). With the advancements of HTS technologies, the amount of sequencing data is continuously growing (4). These technologies present investigators with the challenge of extracting meaningful statistical and biological information from high-dimensional data. The mathematical and statistical properties of high-dimensionality are often poorly understood or overlooked in data modeling and analysis (5). The ability to embed these textual sequences in a vector-space is an important step towards developing effective analysis methods. One possible approach to do so is to use existing embedding methods from the natural language processing (NLP) world, and adapt them to immunological sequences.

In NLP, the term “embedding” refers to the representation of symbolic information in text at the word-level, phrase-level, and even sentence-level, in terms of real number vectors. “Word embedding” was first introduced by (6). Since then, vector space models for semantics are gradually developing and gaining popularity compared with traditional distributed representations. In 2013, (7) brought word embedding to the fore by presenting the “word2vec” method, which is an NLP-embedding method based on an artificial neural networks, and became the basis for many of today’s NLP applications. While there are countless different applications using the above methods, only few published works have implemented them for biological data analysis. The main ones are ProtVec (8), seq2vec (9) and dna2vec (10), all use the word2vec concept introduced by (7). These studies demonstrate the feasibility of sequence embedding. dna2vec provides experimental evidence that the arithmetic of the embedded vectors is akin to nucleotides’ concatenation, while ProtVec shows that tasks like protein family classification and disordered protein detection are not only feasible using the proposed representation and feature extraction method, but also outperform existing classification methods. This approach opens the door for countless exciting opportunities for future developments.

Here, the described line of work is continued, with a focus on developing an embedding technique for B/T cell receptor HTS data, and using this embedding, along with machine learning abilities, to answer real-life questions in computational immunology. To do so, we created an initial word2vec model for immune system sequence embedding. This model will be referred to as Immune2Vec. We use a bottom-up approach, starting from validating the proposed model on short 3-gram amino acid sequences, continuing to longer, complementary determining region 3 (CDR3) sequences, and finally to the full-scale problem of entire immune receptor repertoire representation. Such an approach can pave the way to a wide field of computational immunology applications, which can also exploit the countless on-going developments in the field of machine learning and NLP methods, for different types of high-dimension immunological data analyses.

## The Proposed Approach and Applications

**Figure 1:**
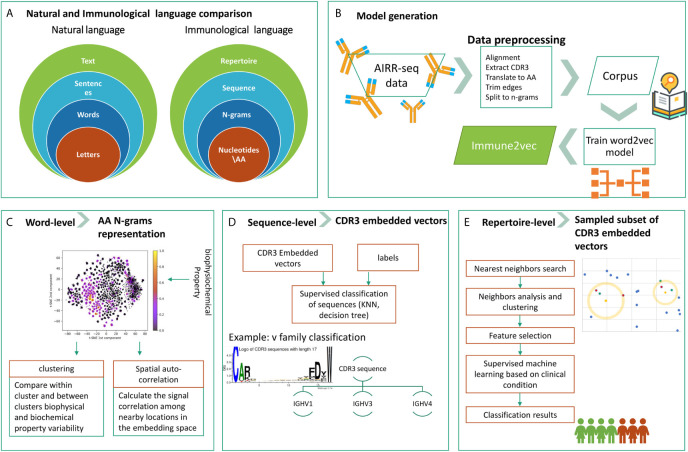
The research structure and workflow. **(A)** The analogy between natural language and the immunological language, on which we base our research. **(B)** The steps of Immune2vec model generation, described in details in the *Methods* section. **(C)** Word-level implementation of Immune2vec on amino acid 3-grams **(D)** Sequence-level classification on CDR3 embedded vectors, classifying them according to the IGHV family of the adjacent IGHV sequence. **(E)** Repertoire-level classification approach based on a nearest neighbors approach presented here. Created using the Weblogo tool (15).

The overall goal of this research is to develop a methodology to embed BCR sequences in a real vector space using methods from NLP. We rely on the evident analogy between immune receptor sequences and natural language, and the applicability of natural language processing methods to immune receptor sequences, to address open questions. The above analogy is illustrated in **Figure 1A**. In this context, the smallest units in natural language are the letters, which correspond to amino acids in immune receptor data. The letters compose words, and several amino acids compose an n-gram. Multiple words produce a sentence, whose parallel in immunology is a receptor sequence. Texts are parallel to repertoires. The main differences between the two are that first, in NLP words have varying lengths, and second, the text level in NLP assumes that sentences are ordered whereas in immune receptor repertoires, there is no such order. The model generation phase, described in **Figure 1B**, takes as an input a large corpus of “text”, in our case AIRR-Seq data, and produces the “Immune2vec” model. This enables transformation of the data to vector space. Based on the described diagram, here we use a bottom-up approach to test immune2vec following the representations in three levels:

1. **Word**: we consider a word as a three amino acids sequence, aka 3-gram. The first step focuses on showing how the embedding approach manages to represent and capture some of the biochemical and biophysical properties of the 3-gram, as described in high-level in **Figure 1C**.
2. **Sentence**: as mentioned, sequences are analogous to sentences in natural language. As **Figure 1D** shows, CDR3 sequences are considered as sentence-level representations. As a proof of concept of the ability to embed sequences in ℝ*^N^*, the real-space vectors that represent the CDR3 sequences are used as input to classification algorithms, with the aim of classifying the sequences according to their corresponding IGHV families of the adjacent sequences.
3. **Text**: representing an immune system repertoire of a subject is a major challenge. It requires finding a proper way to represent a complete immune receptor repertoire, which allows answering complex questions about an individual. For example, stratification of repertories according to a given clinical condition. Here, we provide promising proof of concept results for this, as seen in **Figure 1E**.

## Methods

### Data Sets and Models

The data sets used in this research are:

- **DS1** contains 28 individuals from a study about HCV: 7 healthy individuals as a control group (C), 10 chronically infected (CI) individuals, and 11 spontaneous clearers (SC) of the virus (11). Total number of sequences: 1.3 million BCRs and 1.7 million TCRs.
- **DS2** contains 100 individuals. It is the largest data set, to date, of naive B cell receptor repertoires. It contains 48 healthy control (HC) individuals and 52 patients with Celiac disease (CD). Total number of sequences: 2.96 million (12).
- **DS3** contains the BCR repertoires of three Personal Genome Project volunteers (G.M.C., I.B., and F.V.), at 10 time points along their seasonal influenza vaccination, in order to test the immune response to this vaccine (13). The data set contains 30 samples and 1.64 million sequences.
- **DS4** is a combinatorial synthetic data set of all possible amino acid 3-grams combinations of the 20 common amino acids, producing a total number of 3^20^ = 8000 sequences.
- **DS5** contains BCR repertoires from 13 individuals. Three healthy individuals and 10 infected with COVID-19. Total number of sequences is 7.9 million (14).
- **DS6** contains 1.77 million sequences randomly sampled from DS5.

The above data sets were used for data analyses, classification processes, and to create the following models:

- **IVM1** - An Immune2vec embedding model constructed from a corpus of BCR CDR3s from DS1.
- **IVM2** - An Immune2vec embedding model constructed from a corpus of BCR CDR3s from DS2.
- **IVM3** - An Immune2vec embedding model constructed from a corpus of BCR CDR3s from DS5.
- **IVM4** - An Immune2vec embedding model constructed from a corpus of BCR CDR3s from DS6.
- **IVM5** - An Immune2vec embedding model constructed from a corpus of TCR CDR3s from DS1.

### Immune2vec Model Generation

**Figure 2:**
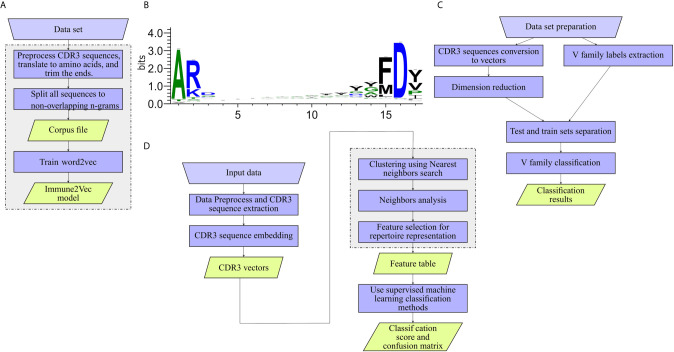
Work flows applied to the different levels. **(A)** Training Immune2Vec. **(B)** Applying Immune2Vec for sequence level classification. **(C)** Applying Immune2Vec to repertoire level representation. **(D)** CDR3 sequence logo for 17 amino acids. Created using the Weblogo tool (15).

The workflow of model generation is shown in **Figure 2A**.

• **Data set**
The first step in creating the model is building an adequate corpus for word2vec training. Selecting the data set is critical to the context analysis, and must be chosen carefully to reflect the type of data we plan to analyze, but not to over-fit it. All our models are generated from amino acid sequences. Building a model based on nucleotide sequences is also possible, but is beyond the scope of this study.
• **Preprocess CDR3 sequences and translate to amino acids** CDR3 sequences were extracted from the data set, and translated to amino acids using the Python Bio.Seq package (16). CDR3 falls at the joint between the V and J segments, and in the heavy chain it is partly encoded by the D gene segment. The diversity of CDR3s is significantly increased by addition and deletion of nucleotides in the formation of the junctions between the gene segments. The final CDR3 begins and ends with a short almost-constant sequence (see **Figure 2B**). In models 1-3, the common amino acids are ignored by *trimming* 2 amino acids from the beginning of the sequence and 3 from the end.
• **Split all sequences to non-overlapping n-grams**
As suggested by (9) in seq2vec, two ways of processing can be employed on the sequences, overlapping and non-overlapping. A non-overlapping method was used throughout this research. Compared to the structure of natural languages, each n-gram now represents a word, and each sequence is a sentence (as described in **Figure 1A**).
• **Corpus file**
The term “corpus” refers to the collection of words extracted from all documents. In our case, the corpus is a file containing all the sequences generated by the non-overlapping method.
• **Adapt word2vec to BCR CDR3 amino acid sequences**
An unsupervised training method composed of a shallow two-layer neural network was used, as described in (17). The algorithm has two options of continuous bag-of-words (CBOW) or skip-gram. CBOW predicts the targeted word given the context, while skip-gram predicts the context given the targeted word. Here, we used skip-gram for all presented results. The model is trained to predict the “context” surrounding a given word, on a corpus built only from the CDR3 amino acid sequences, completely independent of the labels of the data. The labels are only used during the classification part.
• **Model hyper-parameters**

The model contains several hyper parameters that affect the result and must be taken into account. In particular:

- Window size: determines the window surrounding a word for context analysis. Since our method is based only on CDR3 sequences, which are relatively short, we chose a large window of 25 words such that an entire CDR3 sequence is incorporated into the context window.
- Dimensionality: the number of dimensions representing each word in the corpus. The typical number of dimensions embedded by the model is between 50 and 600, as suggested by (7) who show the impact of the vector’s size on accuracy for the following values: 50, 100, 300, 600. Here, we used a vector size of 100, with additional reduction techniques in the different downstream analyses of each section.
- n-grams size: the n-grams size determines the length of the constant n-grams that the data is split into. The value we chose is 3 amino acids, composed originally of 9 nucleotides. This number affects the vocabulary size, which is 20*^n^* ^=3^ = 8000. Choosing a large number causes data loss, since the model does not observe all existing combinations, and sequences that contain these combinations are not embedded. In our case of 3-grams, less than 1% of the data is not present in all corpora.
- Sequence trimming: indicates whether to trim the sequences from one or both ends before training the model. We use the same sequence trimming values while building the model as while applying it to sequences. The trimming values used for each part are indicated above.
- Minimal frequency: words that appear fewer times than this value are ignored in the model training. We used a value of 2, meaning the n-gram has to appear at least twice to be part of the model.
- **Trained model**

The trained model includes the key function called *“model.to_vec*()*”*. This function allows conversion of any sequence to its n-dimension vector, assuming all 3-grams composing the sequence appeared in the training data set. For a sequence composed of several n-grams, the vector is a weighted average of n vectors. Meaning, *n* new sequences are generated from a given sequence by shifting the starting point n times from the beginning of the sequence. For example, for the sequence

Seq: ASLEMATIEDAA

and *n* = 3, three new sequences are generated and translated to vectors:

Sub−seq1: ASLEMATIEDAA→vec1

Sub−seq2: SLEMATIED→vec2

Sub−seq3: SLEMATIEDA→vec3

These vectors are summed and normalized by the number of words of all 3 sequences, so:

$$Vec(Seq)=\frac{vec1+vec2+vec3}{11}$$

- **Fitting hyper parameters of the ML classification model**
- Logistic regression (sections *Stratifying HCV-Specific B-Cell Repertoires Using Immune2vec* and *Corpus Effect*). The logistic regression classifier is implemented with the l2 penalty. Repeated cross validation on the train set was used for selecting the optimal regularization parameter *С* = 0.003.
- k-Nearest neighbors (see the section *BCR CDR3 Sequence-Level Representation Enables Inference of the IGHV Gene Family of the Adjacent Sequence*) - *k*- nearest neighbors algorithm is implemented with the number of neighbors *k* = 3.
- Decision Tree (see the section *BCR CDR3 Sequence-Level Representation Enables Inference of the IGHV Gene Family of the Adjacent Sequence*) - we did not optimize the model hyper parameters and used the defaults provided by the python sklearn package.
- Random forest (see the section *BCR CDR3 Sequence-Level Representation Enables Inference of the IGHV Gene Family of the Adjacent Sequence*) - we did not optimize the model hyper parameters and used the defaults provided by the python sklearn package.

### Word-Level Representation

#### Obtaining Amino Acids Properties

The first building block of the research is validation of the word-level representation model. This validation includes an analysis of 3-grams representation. The vector representation in 2-dimensional space is compared to known properties of these sequences, and spatial auto-correlation methods are applied to validate that the vector representation manages to capture some biologically meaningful data.

Multiple amino acid biophysical and biochemical properties can be obtained using the “alakazam” package in R (18). The following properties were extracted and are used in this section:

- “Gravy”: grand average of hydrophobicity.
- “Bulkiness”: average bulkiness.
- “Polarity”: average polarity.
- “Aliphatic”: normalized aliphatic index.
- “Charge”: normalized net charge.
- “Acidic”: acidic side chain content.
- “Basic”: basic side chain residue content.
- “Aromatic”: aromatic side chain content.

#### Clustering in 2-Dimensional Space

**Figure 3:**
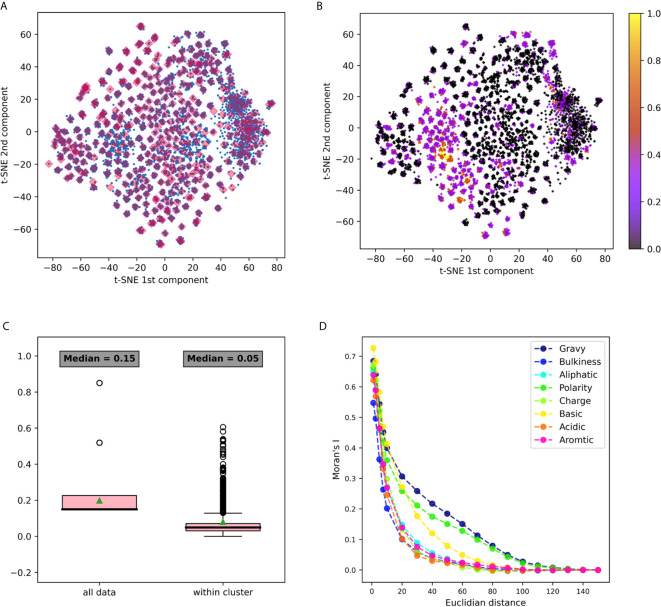
3-gram embedding analysis using several tools **(A)** 3-grams embeddings divided to clusters using k-means clustering **(B)** The same embedding whereeach point is colored according to its basic property value. **(C)** A box plot describing the distribution of basic property distances among all the points, vs. its distancedistribution in each cluster. Comparing distances between all data to the distances within clusters using the Mann Whitney test yielded a p value <10-20. **(D)** Moran’s index spatial auto-correlation analysis of properties in the embedding space.

Several clustering methods on the 2-dimensional space with Euclidean distance were implemented in this section. The first is k-means unsupervised clustering. The clustering was done with a depth of 2, using ***n* = 20**, meaning that the space is divided into 20 clusters, and each cluster is divided again to 20 sub-clusters, creating a total of k=400 clusters. There are a total of 8000 points, thus, on average, each cluster consists of 20 data points (with a standard deviation of 8.5). The centers of these clusters are shown in **Figure 3A**. The second method is k-means with different parameters, dividing the space to 6 clusters followed by another divide to 30 sub clusters, creating a total of 180 clusters. The 6 and 30 parameters were selected based on silhouette score optimization.

#### Variance Calculation

After clusters are defined, our goal is to understand the correlation between the clusters and the biological properties. From **Figure S1** there appears to be a visual correlation, such that clusters tend to have a small in-cluster variance. To quantify this observation, two methods were used based on pooled variance comparison or distance distribution comparison. In the first method, a comparison between the within-cluster variance and the general variance is performed. The within-cluster variance is calculated using the pooled variance method. The pooled variance is a method of estimating variance of different populations, when the populations may vary in size and mean, but share the same variance. Pooled variance computation: if the populations are indexed *i* = 1,.,*k*, then the pooled variance $s_{p}^{2}$can be computed by the weighted average

$$s_{p}^{2}=\frac{\Sigma_{i=1}^{k}(n_{i}-1)s_{i}^{2}}{\Sigma_{i=1}^{k}(n_{i}-1)}=\frac{(n_{1}-1)s_{1}^{2}+(n_{2}-1)s_{2}^{2}+\cdots +(n_{k}-1)s_{k}^{2}}{n_{1}+n_{2}+\cdots +n_{k}-k}$$

where *n_i_* is the sample size of population *i* and the sample variances are

$$s_{i}^{2}=\frac{1}{n_{i}-1}\sum_{j=1}^{n_{i}}(y_{j}-\bar{y_{i}}{)}^{2}$$

In the second method distances in terms of biophysical or biochemical properties are calculated between all vectors within each cluster, and this distribution is compared to the distribution of the same distances between all the vectors in the data. For each property we show a box plot of the distance of this property between all data points *vs*. the distance of the property within each cluster. Use of (*n_i_* – 1) weighting factors instead of *n_i_* comes from Bessel’s correction.

#### Spatial Auto-Correlation Quantification Using Moran’s Index

Spatial auto-correlation is characterized by a correlation in a signal among nearby locations in space. Using this method, we dismiss the clustering effect on the results, by calculation the spatial auto-correlation in the entire 2D embedding space. Moran’s Index, or Moran’s I, (19, 20) quantifies the global spatial auto-correlation in an attribute *y* measured over *n* spatial units, and is given as:

$$I=\frac{n}{S_{0}}\frac{\Sigma_{i}\Sigma_{j}z_{i}w_{ij}z_{j}}{\Sigma_{i}z_{i}z_{j}}$$

where *w_ij_* is the spatial weight matrix, $z_{i}=y_{i}-\hat{y}$, and *S*_0_ = Σ_*i*_ Σ_*j*_ *w*_*ij*_. Moran’s I is used here to evaluate the spatial auto-correlation between amino acids biophysical and biochemical properties relative to their location in the 2-dimensional space. The implementation includes several steps:

1. Choosing a list of distances *d*_1_ *,d*_2_, …*,d_m_* that represents the whole range of distances between points in space.
2. Constructing the spatial weights matrix *w_ij_*. In our case, a weight of 1 was given to two points with a distance smaller than *d_x_*, and 0 otherwise.
3. Calculating Moran’s index for each property and each distance.
4. Analyzing the results, where the expected value under the null hypothesis of no spatial auto-correlation is 0 for large sample sizes as in our case. Positive values indicate positive auto-correlation.

The implementation of this section was done using the python pysal package by (21).

### Sequence-Level Representation Using V-Family Analysis

In this part we focus on one application of sequence-level representation in a classification problem. We chose to classify IGHV families using CDR3 sequences. The models used in the analysis are IVM1 and IVM2. As illustrated in **Figure 2C**, the work flow for IGHV family classifier is composed of:

1. Data preparation – the required output is one file containing (at least) the trimmed CDR3 translated to amino acids, and the V Call. The 3 most common IGHV families were chosen from the data - IGHV1, IGHV3 and IGHV4. To avoid bias, the same number of samples was chosen from each family. As indicated, the sequences were trimmed from both ends. As can be seen in the sequence logo images in **Figure 2B**, the three first amino acids of most sequences are “AR”, and the four last acids are “FD-”, these sub-sequences were removed.
2. Generate vectors from the data set by applying the trained Immune2vec model.
3. Reduce vector dimensions – PCA was used in this section.
4. Use R to extract IGHV family labels using the “getFamily” function from the “alakazam” package by (18).
5. Split the data to test and train data sets. In the train set, each CDR3 sequence was labeled with the corresponding IGHV family of its original VDJ sequence. The size of the train set is 75% of the data, and the size of test set is the remaining 25%.
6. Use different classifiers to distinguish between families and show the results. In this case, the two classification methods used were decision tree and k-nearest neighbors. The classifiers are optimized to the train set, and tested on the independent test set. The classifiers are applied using the python “Scikit-learn” package (22).

### Repertoire-Level Representation

#### Work Flow Description

The general work flow for this part is shown in **Figure 2D**, with each block in the diagram described below.

**• Input data**
For the repertoire-level classification we used 10 CI and 10 SC samples from DS1. For the sequence embedding step we tested the model with the following corpora: data sets DS1, DS2, DS5, and DS6, as described in the section *Data Sets and Models*.
**• Data Preprocess and CDR3 sequence extraction**
Raw reads were filtered in several steps to identify and remove low quality sequences, as described in (23). From each sample, CDR3 sequences were extracted and translated into amino acids using the Python Bio.Seq package by (16). Then, common amino acids from the sequences were trimmed as described.
**• CDR3 sequence embedding**
At this point, Immune2vec was applied to the CDR3 sequences, producing a d-dimensional vector representation for each sequence, with *d* = 100.
**• CDR3 vectors**
The output of applying the model to all sequences, is a matrix of [*numberofsequences*×*d*] size, where each row *r_i_* represents a sequence *i* and each column *С_j_* represents the value of the embedding in dimension *j*.
**• Clustering of Vectors Across all Samples**
Vectors were first grouped according to their V-gene, J-gene, and CDR3 length. For each group, the difference between each pair of vectors was calculated by Euclidean distance. Hierarchical clustering by a complete linkage method was applied and sequences were clustered by a distance threshold. Setting this threshold for the complete linkage presented a new free variable of the model. In (11) and (24) the distance threshold was 85% similarity in hamming distance. In the embedded vector space we used Euclidean distance, and for determining the threshold distance we used the following heuristic: for each set of vectors grouped by their V-gene, J-gene, and CDR3 length, we computed standard deviations for each dimension independently yielding an “std vector”. The threshold used was 0.65 times the Euclidean norm of the “std vector”. As an additional quality control step, sequence clusters for which more than 90% of sequences came from a single sample or clusters containing less than 10 sequences were removed.
**• Extracting features**
As described in (11), the relative frequency of all clusters was calculated for each sample. The sum of frequency squares was calculated for each clinical group. B-cell clusters containing vectors for which the sum of frequencies in SC was greater than the corresponding sum for CI by more than 0.5 were selected. Only clusters with sequences originating from more than one sample, and sequences that were observed in more than one raw read were used.
**• Building feature tables**
Once the features are selected, the information is processed to a feature table, a basic [*N*×*M*] structure with each raw representing a subject and each column representing a cluster number. The value of each cell in the table is the square of the number of sequences belonging to each subject in each cluster. This structure can be used for standard machine learning approaches for the purpose of data classification.
**• Feature elimination**
Feature elimination was performed by a random forest model, choosing the most informative 18 features.
**• Classification model**
Logistic regression with an L2 regularization penalty was applied to these 18 remaining features.

The source code for the implementation can be found in https://bitbucket.org/yaarilab/immune2vec_model/src/master/.

## Results

### Word-Level Representation of 3-Grams

The first step we took to evaluate Immune2vec’s performance on immunological data was to show that the embedding captures immunological properties of the data, as these properties are not part of the model’s training. In general, the analyses presented throughout the manuscript are divided into three steps:

1. Pre-processing AIRR-seq data and extracting the core of the amino acids’ CDR3s.
2. Constructing an embedding model (Immune2Vec), and using it to embed AIRR-seq data.
3. Analyzing the *R^N^* vectors embedded by Immune2Vec at the n-gram, sequence, and whole repertoire levels, using machine learning (ML) methods.

The goal of this section is to test whether unsupervised training of the embedding step (Immune2Vec) preserves the biological properties of all possible amino acid n-grams. As can be seen in this section, vectors corresponding to different n-grams cluster in the embedded space according to the bio-physico-chemical properties of their amino acid n-grams.

The first dataset analyzed was DS1, containing Hepatitis C Virus (HCV) infected individuals and healthy controls (11). It was pre-processed, and relevant CDR3 sequences were extracted, translated to amino-acids and split to n-grams using the “non-overlapping” method. IVM1 is the embedding model that was constructed from this data and that was used in this section. More details about the model creation flow can be found in the *Methods* section (section *Immune2vec Model Generation*). To test the described model, a combinatorial data set of 3-grams, DS4, was used (see details in the section *Data Sets and Models*). Immune2vec was applied to all 8000 3-grams, embedding them to a 100 dimensional space reduced to two dimensions using t-SNE (25). The result of this process is plotted in **Figure 3A**, where each point in the 2-dimensional space represents one of the 8000 3-grams in DS4. Independently, the following biochemical and biophysical properties of each 3-gram were extracted: hydrophobicity, average bulkiness, aliphatic index, average polarity, net charge, basic, acidic, and aromaticity (see the section *Obtaining Amino Acids Properties* in *Methods*). Then, all 3-grams composing DS4 were projected into 2-dimensions using t-SNE, and were colored according to the value of each property. An example of the “basic” property colored plot is shown in **Figure 3B**. The other properties plots can be found in **Supplementary Figure S1**. This visualization demonstrates several interesting observations. First, it shows that the 2-dimensional space can be divided into dense clusters, where the values of the properties are similar (**Figure 3C**). Second, it shows that spatial auto-correlation, i.e., a correlation between properties and their location in the 2-dimensional space, exists (**Figure 3D**).

#### Biophysiochemical Properties Are Homogeneous Within Clusters

The t-SNE dimension reduction method does not preserve distance or density between points, thus tends to cluster points with high affinity during the process. The 3-grams 2 dimension representations were assigned to clusters by k-means unsupervised clustering, as explained in the section *Clustering in 2-Dimensional Space*. All points were divided to 400 clusters, each cluster consisted of an average of 20 n-grams (with a standard deviation of 8.5). These clusters are shown in **Figure 3A**. After clusters were defined, we sought to explore the level of property homogeneity within and between the clusters. To calculate property-specific variance we used the property measurements from the pre-embedded n-grams. The property extraction method is explained in detail in the section *Obtaining Amino Acids Properties*. **Figure 3B** and **Supplementary Figure S1** visualize this within cluster homogeneity. To quantify this effect, two methods were applied:

1. A comparison between within cluster and between cluster variability. The within cluster variability is calculated using the pooled variance method (see *Variance Calculation*). The results of this method are shown in **Table 1**. **Figure 3C** shows box plots of the distances between n-grams, once for n-grams within the same cluster and once for all n-grams in the data. **Supplementary Figure S2** shows analog plots for all the examined properties. This confirms that 3-grams cluster into groups, and that the similarity of the properties within each cluster is relatively high. This result indicates that the suggested embedding successfully captures meaningful features of the data, such as the bio-physico-chemical properties of the n-grams. We also tested different clustering approaches and obtained similar outcomes, strengthening our confidence in the robustness of these results.
2. Spatial auto-correlation. To ensure that the clustering parameters did not affect our results, we quantified the spatial auto-correlation of the entire 2D space using Moran’s Index. As explained in the section *Spatial Auto-Correlation Quantification Using Moran’s Index* in *Methods*, spatial auto-correlation is characterized by a correlation in a signal among nearby locations in space. In this case, the signal is the value of the property of each n-gram. **Figure 3D** shows the results of Moran’s Index analysis for each chosen property, with distances between points varying between 1 and 160. A positive auto-correlation exists between nearby n-grams, and as the distance increases, the correlation decreases to 0. In other words, a short distance in the [*x, y*] space is translated to a small variance in the 3-gram property. When using word2vec on natural language, words with similar meanings have similar vectors and are represented near each other in space. Correspondingly, we show that in terms of biological meaning, similar n-grams are also preserving the same space similarity.

**Table 1:** Amino acids properties distribution.

Property name	General variance	K-means (400 clusters)		K-means (180 clusters)	
		In-cluster variance	Ratio	In-cluster variance	Ratio
Gravy	2.83	1.08	1.37	2.1	2.3
Bulkiness	6.8	3.6	1.9	4.06	1.7
Polarity	2.29	0.91	2.5	1.14	2.0
Aliphatic	0.55	0.24	2.3	0.3	1.8
Charge	0.6	0.25	2.5	0.33	1.8
Acidic	0.03	0.01	2.2	0.02	1.8
Basic	0.04	0.01	3.0	0.02	1.9
Aromatic	0.05	0.02	2.3	0.03	1.8

The table compares the general variance of each property for all sequences with the in-cluster variance, and the ratio between them, for three kinds of clustering methods. A high ratio indicates a strong similarity of the property within the cluster.

### BCR CDR3 Sequence-Level Representation Enables Inference of the IGHV Gene Family of the Adjacent Sequence

**Figure 4:**
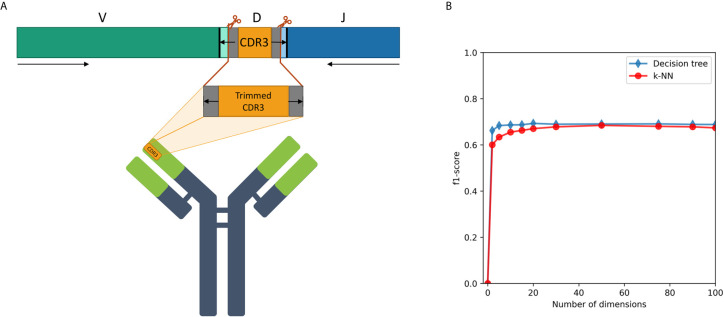
**(A)** A description of the trimmed CDR3 sequences from the Ig heavy chain germline locus, used for the research. **(B)** F1-score of IGHV family classification based on CDR3 sequences using decision tree and kNN methods.

We examined a sequence-level classification problem, as an intermediate step between word-level and whole immune repertoire analysis. As shown schematically in **Figure 4A**, CDR3 falls at the junction between the V and J segments, and in the heavy chain it is partly encoded also by the D segment. It has no overlap with the V segment, thus being able to classify IGHV family based on its corresponding CDR3 sequence will be a novel observation.

First, we extracted CDR3 sequences, trimmed and embedded them in real-space by immune2vec. We assigned a label to each sequence with its IGHV family as inferred from the adjacent 5’ sequence. We then split the data to test and train sets. The training process included optimizing the model parameters on the train set, and the resulting model was assessed on the test set. **Table 2** summarizes the results of IGHV family classification using two types of common classifiers - decision tree and k-NN, on 10-dimensional data. The classification was done on the three most common IGHV families in the data set: IGHV1, IGHV3, IGHV4. In order to avoid biases caused by unbalanced data, an equal amount of sequences from each data set were sampled, without replacement (see right column in **Table 2**). The IGHV family distribution of all the data sets is shown in **Supplementary Table 1**.

**Table 2:** IGHV family classification based on CDR3 sequence using Decision Tree (DT), Random Forest (RF) and K-nearest neighbor (KNN).

Model (corpus)	Data set	DT f1-scoree	RF f1-score	KNN f1-score	Number of samples per family
IVM1	DS1	0.65	0.67	0.58	150K
IVM1	DS2	0.50	0.51	0.46	300K
IVM1	DS3	0.67	0.69	0.61	100K
IVM2	DS1	0.65	0.66	0.64	150K
IVM2	DS2	0.50	0.52	0.46	300K
IVM2	DS3	0.67	0.69	0.59	100K

An association between the trimmed CDR3 and its adjacent V-family exists, and this association is preserved after Immune2vec embedding. IVM2 shows slightly better results, most likely since it is based on a much larger corpus file. The effect of the number of dimensions used by Immune2vec on the classification process was tested by using IVM2 with DS3. The same classification process was performed on a varying number of dimensions. The results for IGHV family classification are shown in **Figure 4B**. From this analysis we conclude that for the task of sequence level classification, it is sufficient to use 30 dimensions. The analysis of this part demonstrates that our representation successfully captures meaningful biological data not only at the word level, but also at the sequence level, which is analogous to sentences in natural language.

### Stratifying HCV-Specific B-Cell Repertoires Using Immune2vec

After gaining confidence in the word and sentence level representations, the next step is repertoire-level representation. The challenge here is to represent accurately an entire immune repertoire, and to use it as an input for machine learning applications such as classification. To investigate whole immune repertoires, we embedded all CDR3 amino acid sequences of a repertoire using Immune2vec. To this end we used IVM3 (see *Data Sets and Models*), and applied it to DS1, a data set composed of three clinically distinct cohorts: healthy controls (HC), chronically infected (CI) individuals with HCV, and spontaneous clearers (SC) of hepatitis C virus. Since there are only seven HC samples, here we applied a two class classification for the CI and SC cohorts. CDR3 sequences were embedded into ℝ^100^, yielding a matrix of size [*number of sequences ×* 100] where each row *r_i_* represents a sequence *i* and each column *C_j_* represents the value of the embedding in dimension *j*(*j* ∈ {1…100}). To characterize whole repertoires using CDR3 embedded vectors we applied an analogous clustering approach to the one used in (11) and (24). Vectors were first grouped according to their V-gene, J-gene, and CDR3 length. For each such group, the difference between each pair of vectors was calculated using a Euclidean distance in the embedded space. Hierarchical clustering by a complete linkage method was then applied and clusters were defined by a maximum distance. Given these clusters, each repertoire was characterized using the relative usage of each cluster in it. Following this, a feature selection step was applied using random forest to select the most informative 18 features. These were the input of a logistic regression step with L2 regularization penalty (for more details see methods *Repertoire-level representation*).

**Figure 5:**
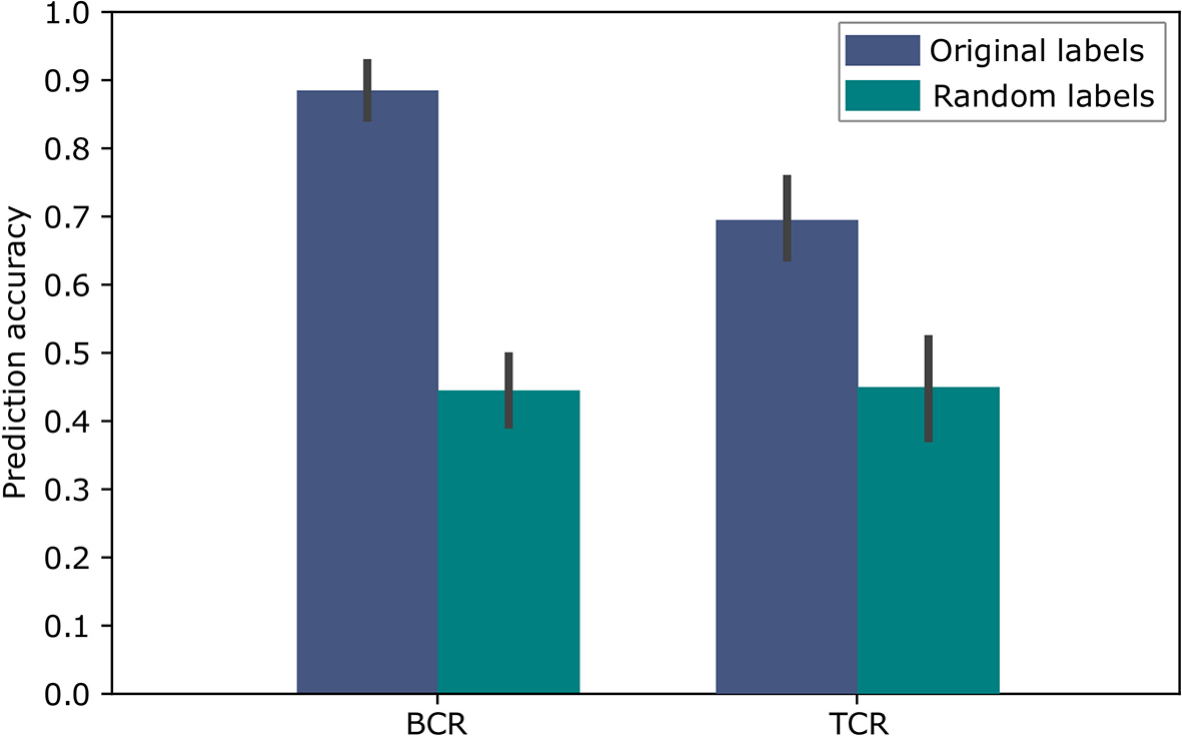
Accuracy of the SC-CI BCR and TCR repertoires classification. For validation purposes, the model was trained and applied on randomly labeled data.

To evaluate the above approach we used repeated cross validations with 100 repeats, leaving in each instance two different samples, one of each label, as the test set. The results of this binary classification are shown in **Figure 5** for BCR data and TCR data. These results are significant compared to random assignments of labels on the same data (p-value < 2.2e-16 according to a two tailed Welch t test), and are comparable to the results in (11), which obtained 91% accuracy for the BCR based predictions and 79% accuracy for the TCR based predictions. In (11), the amino acid CDR3 sequences were clustered using the conventional non-embedding approach, while the other details of the classification model and the way it was tested remained the same. The comparison to random labels supplies additional evidence that there was no over-fitting during the process. These results provide a proof of concept showing that BCR and TCR IGHV family gene sequence embedding can lead to meaningful representations, as can be seen in the high classification results. It is the first demonstration of using word embedding for BCR and TCR repertoire classification.

### Corpus Effect

**Figure 6:**
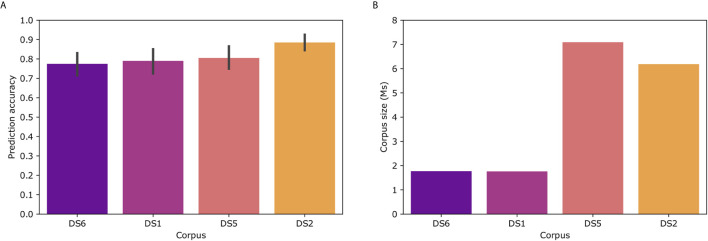
**(A)** Model prediction total accuracy using different data sets as corpora for creating the embedding model. **(B)** Number of sequences in each corpus. DS6 was generated by randomly sampling sequences from DS5.

To examine the effect of the corpus on Immune2vec, we constructed Immune2vec embedding models using CDR3 sequences from different data sets and of different sizes. The results for classification of patients’ clinical status are shown in **Figure 6A**. As can be expected, the size of the corpus affects the accuracy of the model. The best result (89%) was obtained with the DS2 corpus. All models were tested using the same 100 train-test splits, and same ML model. **Figure 6B** shows the size of the different corpora. To examine the effect of the corpus chain, i.e., IGH or TCR*β*, on the model, we compared TCR repertoire classification results, once using a TCR corpus (DS1) for constructing the embedding, and once using a BCR corpus (DS2). As shown in **Supplementary Figure S3** using a BCR corpus for classifying TCR repertoire had an accuracy of 56% compared to 70% when using a TCR corpus. The higher accuracy when using a TCR corpus can be explained by 1) The characteristics of the embedding corpus - in this case using TCR sequences for both the embedding construction and the classification step, 2) The embedding corpus and classification use samples from the same population (DS1) and/or, 3) The embedding step uses the whole DS1, which consists of the training and test TCRs in the classification step. Since in the case of BCR, using DS2 as the embedding corpus leads to a higher prediction accuracy than using DS1 (**Figure 6A**), it implies that the first confounding factor has a stronger effect on the prediction accuracy than the later two.

## Discussion

Biology and medicine are data-rich disciplines. The high throughput measurements used in these fields are complex and require profound knowledge to understand. For this reason, deep learning is well suited to solve problems in these fields, as it is capable of combining raw inputs into features. New deep learning algorithms were developed for a variety of biomedical problems such as patient classification, biological process analysis and treatment methods (26). This study conducts a thorough literature review and claims that although promising advances have been made in the field of deep learning, it has yet to revolutionize bio-medicine or definitely resolve any of the most pressing challenges. Several recent publications focus on deep learning applications for immune system repertoires (27). Some of them focus on similar issues to the ones studied here, like immune receptor repertoire representation. DeepTCR (28) for example, applies sequences embedded using variational auto encoder (VAE) instead of the linguistic approach used in our study (29). also use a VAE approach on adaptive immune repertoires, with the purpose of constructing generative probabilistic models.

This research focuses on developing a methodology to embed BCR cDNA and protein sequences in a real vector space using methods from NLP. The embedding proposed here enables the numerical representation of high-dimensional sequences. As such, it enables the applications of standard tools, such as dimensional reduction and classification to these data. Thus, obtaining meaningful numeric representations of immune receptor sequences, which does not suffer from the curse of dimensionality and can act as input to various machine learning architectures, is necessary. It opens the door to countless applications, developed in the past few years for natural language processing, and widely used in different industries including machine translation, sentiment analysis, auto-completion, and many more, which can be adapted and explored in the medical field for real-life problems.

Several studies specifically applied word embedding to different types of biological data. Medical text analysis was studied by (30–32), and (33); the “DNA2vec” (10) method presents a distributed representation of nucleotide n-grams; methods such as “Splicevec” by (34) and “Gene2vec” by (35) focus on genomic sequence analysis. The work presented in “ProtVec” (8), inspired our approach, as it suggests the use of word2vec method for protein representation. This work shows how such approach performs in tasks like protein family classification, structure prediction and visualization, disordered protein identification, and protein-protein interaction prediction (36). also adapted this approach for the purpose of identifying antimicrobial peptides. As in the case of word2vec and deep NLP models, immune2vec can be effective as a pre-trained embedding layer for deep NN models that use immune sequences as inputs.

In real natural language analysis, one can examine the results of an embedding by simple measures, such as viewing the words that cluster together on a 2-dimensional map, or examining the relations between known words to understand the quality of the model. In biological data, there is more than meets the eye, as there are no semantically meaningful sequences, and deep biological understanding is required to analyze and evaluate the results. In addition, the problem becomes even more complex when trying to represent a wide combination of sequences, such as the entire immune receptor repertoire. The proposed method is first of its kind, involving several steps, multiple hyper-parameters and degrees of freedom.

Bearing in mind these challenges, we built this work in steps, each step designed to raise our confidence in the model, allowing a scale-up in the level of complexity with each result. Following the structure of a natural language, in which words compose sentences and sentences compose whole texts, we examine short sequences of amino acids as words, CDR3 sequences as sentences, and the immune system repertoire as text. This last analogy is not completely accurate, as the order of sentences in books or article is meaningful, while the immune receptor repertoire data is a collection of sequences without biologically relevant order or context. Thus, we did not apply common text analysis tools, such as doc2vec, but preferred to find a way to represent a repertoire by an overall analysis of its set of sequences.

A comprehensive comparison between different AIRR-seq embedding methods that would encompass the many dimensions of AIRR-seq such as the type of chain (IGH, IGK, IGL, TRB, TRA, etc.), type of signal (nucleotide/amino acid enriched motifs, changes in the overall diversity of a repertoire, etc.), DNA library preparation protocol, sequencing technology, etc., is of utmost importance. Such a comparison falls outside the scope of the current manuscript, and is therefore aimed for a future independent study.

Our work demonstrates that sequence embedding using NLP methods, enables low-dimensional representations that preserve relevant information about the sequencing data, such as n-gram properties and sequence gene family classification. We show that this information is meaningful, as we found clinical condition indicators that enable classification of HCV patients. This indicates that the embedding space can be used for feature extraction and exploratory data analysis. At the computational level, first, once our data is correctly transferred to numeric representation, we can exploit the on-going developments from other fields and adapt them to answer biological questions. The field of machine learning and NLP is advancing in a rapid pace, with a plethora of approaches and tools. Second, a corpus file can be generated from non-labeled data, so a large pool of immune receptor repertoire data can be used to build a large corpus for several applications. Furthermore, once a labeled data-set is available, it is easily transformed to numerical representations and processed *via* the suggested pipeline to form a feature table that can be analyzed by multiple approaches, and answer different questions by changing the training labels.

While this work provides encouraging results on immune system representation using NLP methods, there is a lot to be done before the suggested methods can be clinically valuable. First, a large amount of the data should be collected and incorporated into a large corpus, which can be used to train a general immune repertoire model independent of the effects of different conditions and diseases that exist when basing a model on a single data set. Second, a major challenge is exhibited in the clustering and feature extraction phase of repertoire data. If we choose to keep a large number of features, the data becomes sparse and hard to cluster in high-dimensional space, calling for tailored approaches for the type of data presented here. Future work should aim at elucidating the effects of different training data sets and obtaining a better understanding of the feature representations, develop further the repertoire presentation approach, and extend the sequence level representation to include information about the specific antigens that are associated with sets of receptors.

To conclude, Immune2vec embeds BCR and TCR sequences in real vector space, using methods from natural language properties. It shows great advantages, and we hope to continue and further unravel this approach to a simple and robust workflow that can be easily applied to new data sets.

## Data Availability Statement

Publicly available datasets were analyzed in this study. These data can be found here: https://www.ebi.ac.uk/ena/browser/view/ERR2843386-ERR2843427 and https://www.ebi.ac.uk/ena/browser/view/PRJEB26509.

## Ethics Statement

Ethical review and approval was not required for the study on human participants in accordance with the local legislation and institutional requirements. The patients/participants provided their written informed consent to participate in this study.

## Author Contributions

GY and MO-B conceived the project and developed the ML approach. MO-B and BF implemented the algorithms and analyzed the data. GY supervised the project. GY, PP, and MO-B wrote the paper. All authors edited the manuscript. All authors contributed to the article and approved the submitted version.

## Funding

ISF [832/16]; European Union’s Horizon 2020 research and innovation program [825821]. The contents of this document are the sole responsibility of the iReceptor Plus Consortium and can under no circumstances be regarded as reflecting the position of the European Union.

## Conflict of Interest

The authors declare that the research was conducted in the absence of any commercial or financial relationships that could be construed as a potential conflict of interest.
