# Supplementary material for: Immune2vec: Embedding B/T Cell Receptor Sequences in ℝN Using Natural Language Processing
Source: Front Immunol. 2021 Jul 22;12:680687. doi: 10.3389/fimmu.2021.680687 (PMC8340020; doi:10.3389/fimmu.2021.680687)
Supplement: Supplementary file 1 [file DataSheet_1.pdf]

Supplementary Material

1 SUPPLEMENTARY DATA

Data set	IGHV1	IGHV2	IGHV3	IGHV4	IGHV5	IGHV6	IGHV7	Total	Sampled
DS1	195209	52466	580947	266947	39852	10941	2863	1149295	190000
DS2	594872	93271	1400248	732276	119100	20078	8850	2969388	500000
DS3	114766	17482	369089	131634	14863	4582	1510	653975	114000

Table S1. A table describing the distribution of V families in each data set

Data set name	Number of samples	Total number of sequences	Clinical conditions	Related disease	Comments
DS1	28	1.3M BCRs and 1.7M TCRs	C, CI, CD	HCV	Eliyahu et al. (2018)
DS2	100	2.96M	HC, CD	Celiac	Gidoni et al. (2019)
DS3	30	1.64M	Not used	Seasonal influenza	Laserson et al. (2014)
DS4	-	8000	-	-	Combinatorial data set of all amino acid 3-grams
DS5	13	7.9M	Not used	COVID-19	Kuri-Cervantes et al. (2020)

Table S2. A summary of all data sets used in the research

2 SUPPLEMENTARY FIGURES

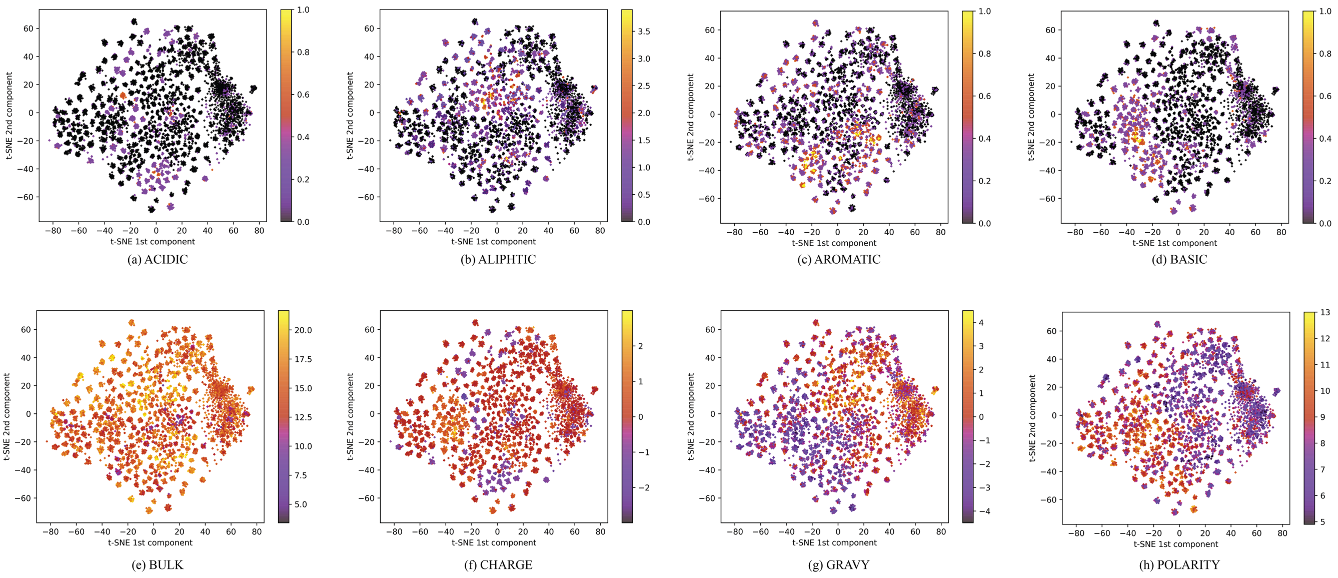

Figure S1. A plot of the 8 examined AA properties, where each point is a 3-gram in 2-dimensional space, and the color corresponds to the value of the specific property.

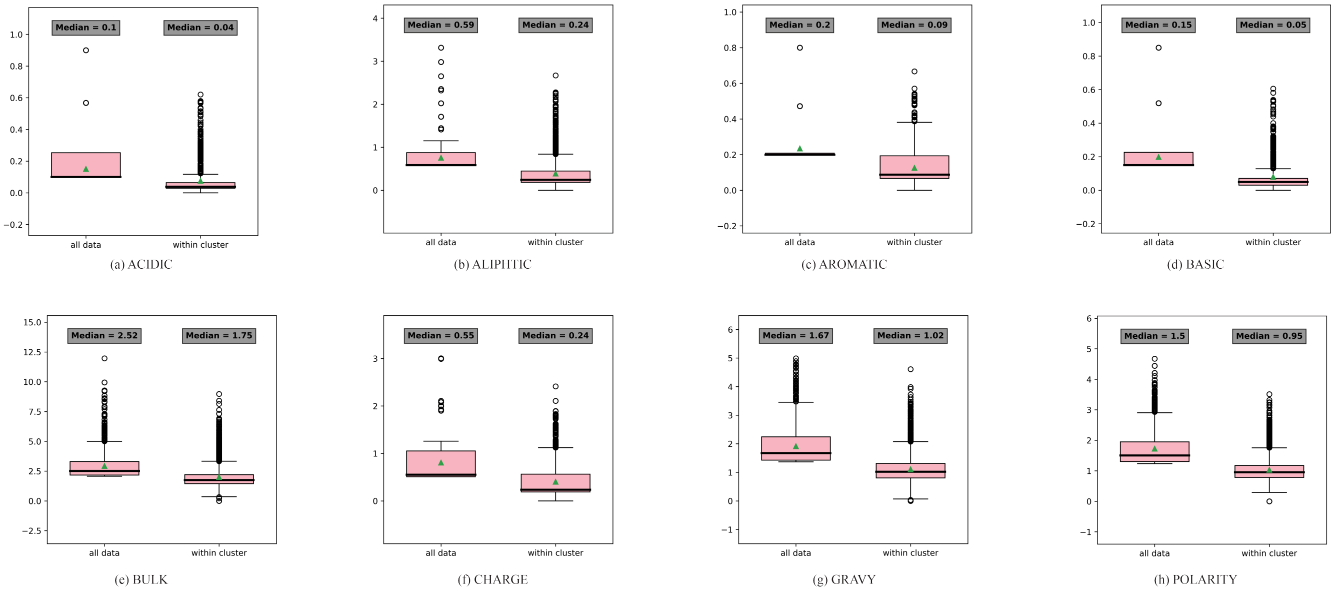

Figure S2. A box plot of the property distance distribution of all examined AA properties, among all points vs. the distance distribution within each cluster. Comparing all data to the distances within clusters of all these properties yielded p values$< 10^{-20}$ after applying the Bonferroni correction for multiple hypotheses.

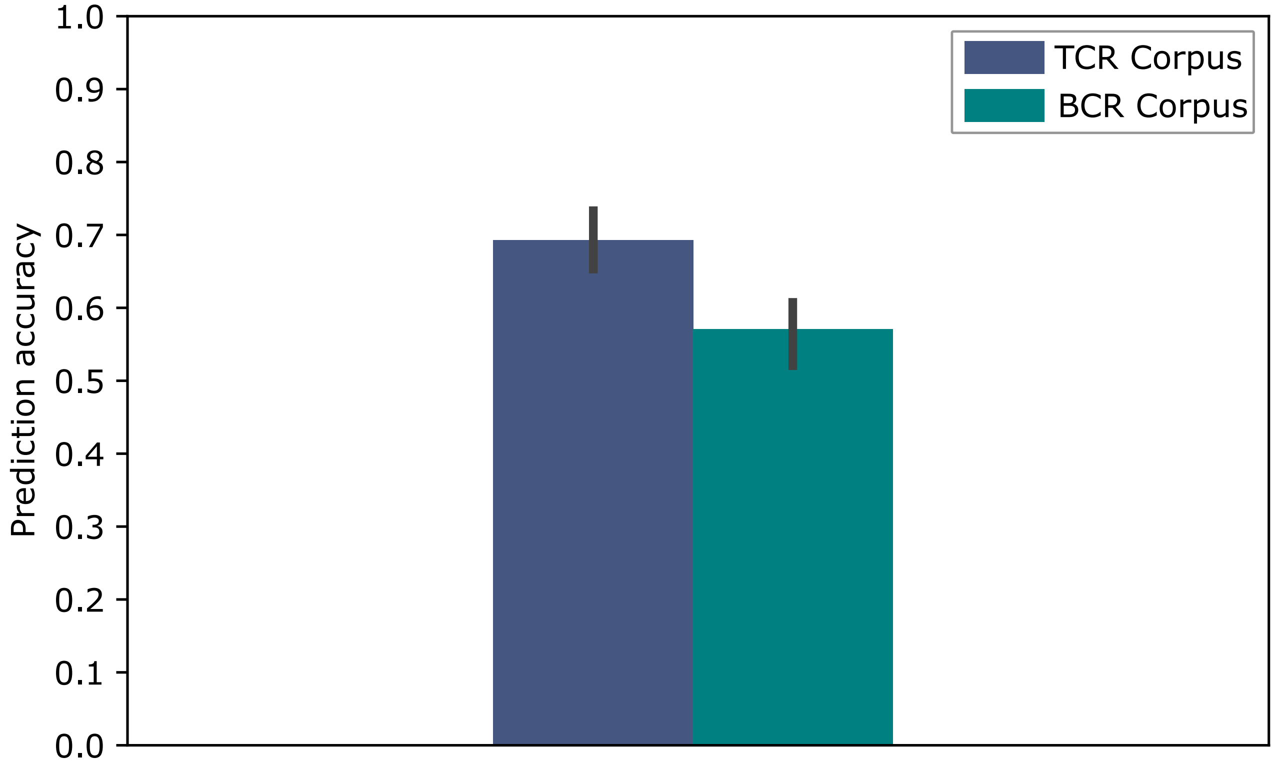

Figure S3. TCR repertoire classification results using different corpora. Prediction accuracy is shown once for a model that was constructed from TCR sequences, and once for a model that was constructed from BCR sequences. The TCR corpus is built from DS1 and the BCR corpus from DS2. Results were obtained by using the same 100 ”leave two out” cross validation folds for both models.

REFERENCES

Eliyahu, S., Sharabi, O., Elmedvi, S., Timor, R., Davidovich, A., Vigneault, F., et al. (2018). Antibody repertoire analysis of hepatitis c virus infections identifies immune signatures associated with spontaneous clearance. *Frontiers in Immunology* 9, 3004. doi:10.3389/fimmu.2018.03004

Gidoni, M., Snir, O., Peres, A., Polak, P., Lindeman, I., Mikocziova, I., et al. (2019). Mosaic deletion patterns of the human antibody heavy chain gene locus shown by bayesian haplotyping. *Nature communications* 10, 628

Kuri-Cervantes, L., Pampena, M. B., Meng, W., Rosenfeld, A. M., Ittner, C. A., Weisman, A. R., et al. (2020). Comprehensive mapping of immune perturbations associated with severe covid-19. *Science immunology* 5

Laserson, U., Vigneault, F., Gadala-Maria, D., Yaari, G., Uduman, M., Vander Heiden, J. A., et al. (2014). High-resolution antibody dynamics of vaccine-induced immune responses. *Proceedings of the National Academy of Sciences* 111, 4928–4933. doi:10.1073/pnas.1323862111
